# Density of predating Asian hornets at hives disturbs the 3D flight performance of honey bees and decreases predation success

**DOI:** 10.1002/ece3.9902

**Published:** 2023-03-28

**Authors:** Juliette Poidatz, Guillaume Chiron, Peter Kennedy, Juliet Osborne, Fabrice Requier

**Affiliations:** ^1^ Environment and Sustainability Institute University of Exeter Penryn UK; ^2^ CIRAD, UMR PVBMT La Réunion France; ^3^ Laboratoire L3i Université de La Rochelle La Rochelle France; ^4^ Université Paris‐Saclay, CNRS, IRD UMR Évolution, Génomes, Comportement et Écologie Gif‐sur‐Yvette France

**Keywords:** *Apis mellifera*, flight performance, image‐based tracking, predator–prey interaction, stereovision, *Vespa velutina*

## Abstract

Automated 3D image‐based tracking systems are new and promising devices to investigate the foraging behavior of flying animals with great accuracy and precision. 3D analyses can provide accurate assessments of flight performance in regard to speed, curvature, and hovering. However, there have been few applications of this technology in ecology, particularly for insects. We used this technology to analyze the behavioral interactions between the Western honey bee *Apis mellifera* and its invasive predator the Asian hornet, *Vespa velutina nigrithorax*. We investigated whether predation success could be affected by flight speed, flight curvature, and hovering of the Asian hornet and honey bees in front of one beehive. We recorded a total of 603,259 flight trajectories and 5175 predator–prey flight interactions leading to 126 successful predation events, representing 2.4% predation success. Flight speeds of hornets in front of hive entrances were much lower than that of their bee prey; in contrast to hovering capacity, while curvature range overlapped between the two species. There were large differences in speed, curvature, and hovering between the exit and entrance flights of honey bees. Interestingly, we found hornet density affected flight performance of both honey bees and hornets. Higher hornet density led to a decrease in the speed of honey bees leaving the hive, and an increase in the speed of honey bees entering the hive, together with more curved flight trajectories. These effects suggest some predator avoidance behavior by the bees. Higher honey bee flight curvature resulted in lower hornet predation success. Results showed an increase in predation success when hornet number increased up to 8 individuals, above which predation success decreased, likely due to competition among predators. Although based on a single colony, this study reveals interesting outcomes derived from the use of automated 3D tracking to derive accurate measures of individual behavior and behavioral interactions among flying species.

## Graphical abstract

In this study, we used 3D image based tracking to analyse the behavioural interactions between the Western honey bee Apis melifera and its invasive predator the Asian hornet, Vespa velutina nigrithorax. We investigated whether predation success could be affected by flight speed, flight curvature, and hovering of the Asian hornet and honey bees in front of the beehive.

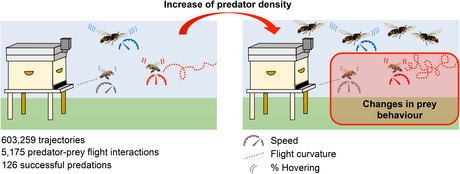

## 1 INTRODUCTION

Movement capacity and foraging performance are key traits in ecology affecting survival and dispersal and have been explored in a large range of animals, like marine predators (Humphries et al., 2010), mammals (Clarin et al., 2013; Sinclair, 1992), birds (Naef‐Daenzer & Keller, 1999), and insects (Capaldi et al., 2000; Holway & Case, 1999; Sumpter & Pratt, 2003). The miniaturization of technology has allowed the development of automated tracking devices (e.g., GPS, Argos; Kissling et al., 2014) surpassing the performance of traditional Movement Capacity Record (MCR) methods. However, automated tracking devices are often restrictive in terms of sample size (1–20 individuals in general) and size of animals targeted (related to the weight of the tracking device). Different tracking technologies can be selected depending on the locomotory mode of the targeted animal (e.g., flight, walk, swim), their environment (e.g., water, air, ground surface), the weight of the tracking device and its optimal attachment to the animal. But such tracking tools require individual manipulation and frequently the addition of extra weight can impact individual behavioral performance (Batsleer et al., 2020).

Image‐based tracking is a good alternative that is increasing in use in animal ecology (Dell et al., 2014). These devices are not invasive as they do not rely on catching the individual nor the attachment of, for example, a microchip/GPS tag, and allow the tracking of several targets simultaneously, enabling the observation of complex behaviors and interactions between multiple individuals (Bozek et al., 2021). Two dimensional (2D) image‐based tracking to study foraging behavior, learning, and/or vigilance of animals is quite common (Noldus et al., 2002; Peters et al., 2016; Wajnberg & Colazza, 1998). However, most animals move in three dimensions (3D). Birds, bats, and flying insects move in 3D in the air, as do fish and sea mammals in the water, which limits the accuracy and precision of 2D data recording. Given the ability to assess the distance from the object in all three dimensions, 3D‐image based tracking can describe adjusted flight/swim behaviors (e.g., speed, curvature, orientation), even if individuals are close to one another (Campbell et al., 2008; Chiron et al., 2015). An additional advantage of 3D‐imaging devices such as stereovision cameras is their ability to recover target positions directly in metric coordinates, as these systems are precalibrated in advance for a specific need (e.g., close focal length, wide field of view). In comparison, traditional 2D image‐based tracking devices (e.g., common cameras) would need the use of a test chart to convert those 2D pixel expressed coordinates to metric coordinates, and would be less accurate with a varying 3rd dimension. 3D image‐based tracking devices have been used to study Malaria mosquito flight, for example (Spitzen et al., 2013), and bat flight patterns in different landscapes (Falk et al., 2014). Stereovision cameras are a tool often used in medicine (Skvara et al., 2013) and engineering (Gao et al., 2011; Huynh et al., 2016), but less in ecology (but see Theriault et al. (2010) and Matzner et al. (2020) for bats and birds, and Rachinas‐Lopes et al. (2019) for water mammals, Chiron et al. (2014) for insects, and more generally Straw et al. (2011)) although benefits are numerous when studying the behavior of 3D‐moving animals.

*Vespa velutina nigrithorax* is an invasive alien hornet in Europe that now needs to be considered among the multiple stressors affecting honey bee survival (Monceau et al., 2014; Requier, Rome, et al., 2019). On top of being a generalist predator of insects, it is capable of predating honey bees in high numbers in front of their hives, demonstrating a specific predation behavior described as “hawking,” when the hornet hovers in front of the hive waiting for its prey (Monceau et al., 2014; Tan et al., 2007). This increasing predation pressure through summer and autumn is leading to heavy honey bee colony losses (between 5 and 80% of colony losses in France, 30% of colony loss in average; Kennedy et al., 2018; Requier, Rome, et al., 2019), via two phenomena. The first one is the direct impact of predation which decreases the number of available foragers in the hive (Tan et al., 2007). The second is “foraging paralysis,” when the honey bee colony stops sending foragers out and there is a consequent decline in incoming food resources (Requier et al., 2020).

Here we applied 3D image‐based tracking to a multipredator–prey relationship, focusing on two model species: the invasive Asian hornet *Vespa velutina nigrithorax* (called “Hornet” in this study) and its prey, the Western honey bee *Apis mellifera* (called “Honey bee” in this study). Using a stereovision camera, we carried out automated processing of 3D image‐based tracks in the field to record 3D‐adjusted behavioral parameters and to focus on specific interindividual interactions. This study aims at understanding the effects of predator (Asian hornet) density on both the flight behavior of predators and prey (honey bees), in a biogeographical context (western Europe) where the prey–predator interaction did not evolve or co‐adapt. First, we developed an automated process to select “scenes of interest,” when both prey and predator are present on the screen at the same time. Such an automated process helps ecologists in video analyses and reduces potential observation bias. Second, we explored flight performances in terms of speed, curvature, and hovering by honey bees and hornets looking for potential drivers of predation success. We assumed that the flight performances of hornets and honey bees differ due to the morphological differences between these species. Moreover, we hypothesized that the predation success could be influenced by predator density due to a disturbance in flight performance of both predator and prey.

## 2 MATERIALS AND METHODS

### 2.1 Automated 3D image‐based tracking system

**FIGURE 1:**
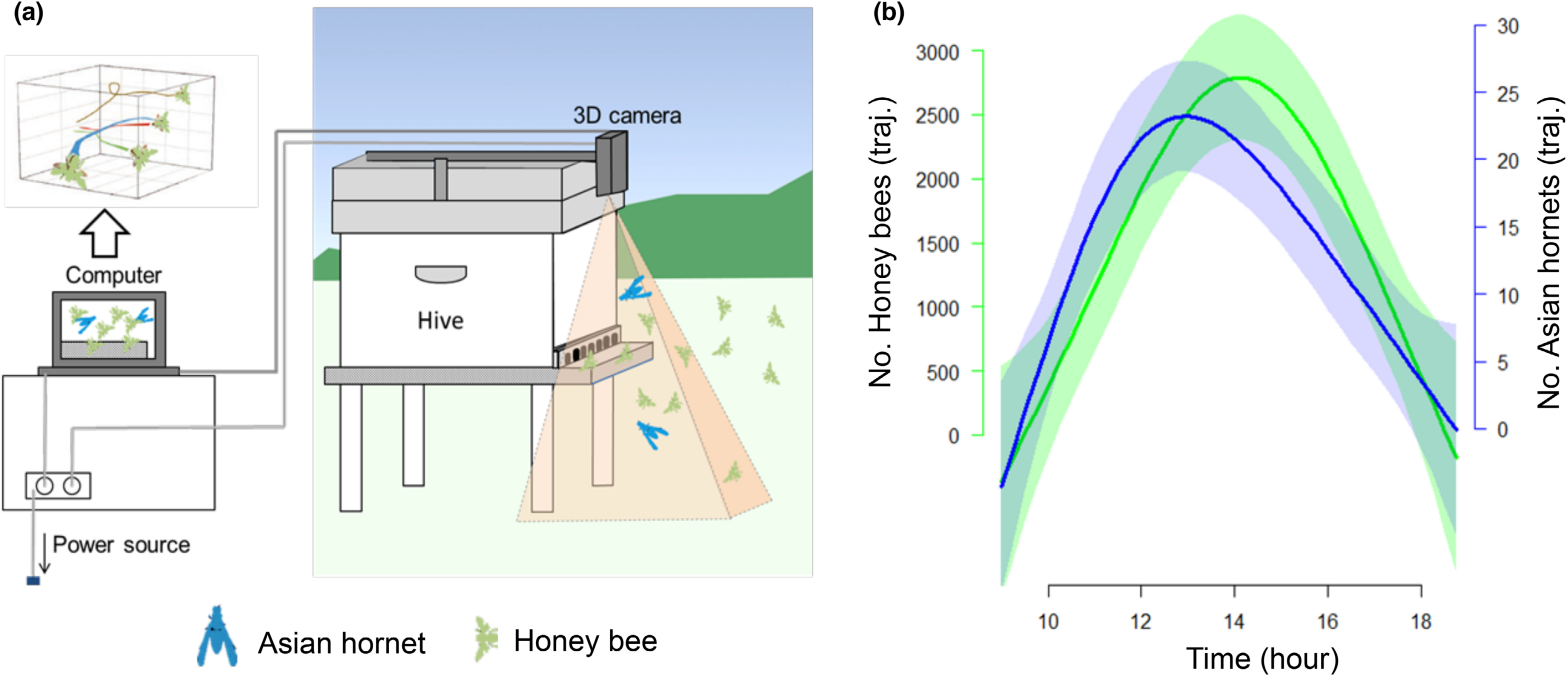
Examining 3D flight performance in honey bees and Asian hornets. (a) Conceptualization of the experimental setup with a stereovision camera fixed over a hive flight board, registering all activity in the volume of air in front of the hive on a computer, which analyses the trajectories of Asian hornets (blue) and honey bees (green). (b) Daily time series of the total number of honey bees (green) and hornets (blue) (trajectories). Lines represent model predictions and shaded areas show the 95% confidence intervals.

A high‐speed stereovision camera (G3 Evo 3, TYZX®; TYZX, 2009; Figure S1) was fixed on the top of a beehive (10‐frame Dadant type) to track the flights of bees and hornets at the beehive entrance (Figure 1a). The stereovision camera was placed 50 cm above the flight board of the beehive to ensure the nontrivial trade‐off between the device intrusiveness (no nearby source of disturbance), the image definition (at least 8 pixels per bee on the flight board), and the observed volume (that must include the 50 cm wide flight board, Figure S1). Software was encoded in the camera hardware to preprocess the trajectories in video data as described in Woodfill et al. (2004). A controller laptop was used to schedule the recordings and to encode the raw RGB‐D videos before being dumped on a NAS (Network Attached Storage). Video surveillance was deemed as a robust and effective method to measure the exact number of honey bees at the entrance of the colony. The experimental beehive was located nearby the town of La Rochelle (France) (46°8’N, 1°8’W), 200 km from where the Asian hornet was first spotted in 2004 (Haxaire et al., 2006; Villemant et al., 2011). The video surveillance was carried out from October 16th to October 25th 2015. In this oceanic climate, the ambient temperature ranged between 6°C and 17°C, wind speed ranged between 11 km.h^−1^ and 39 km.h^−1^, and the relative humidity ranged between 65 and 82%. Video tracking was performed from 9 am to 6 pm over 10 consecutive days, providing a total of 90 hours of recorded activity, corresponding to more than a terabyte of compressed data (i.e., RGB‐D videos above 50 fps). The trajectory detection software was cross‐validated with human observers which gave acceptable cross‐validation assessments with a ‘false alarm’ rate of 0.1954 and a ‘missed detection’ rate of 0.0415 (Chiron et al., 2013).

The monitored beehive was isolated in the apiary (i.e., no other beehives in a 300 m radius) in order to ensure that all observed trajectories belonged to the target hive. The honey bee colony was not bred from any particular genetic strain and was previously caught as a local swarm. In this part of France, the native populations are *Apis mellifera mellifera* (Requier, Garnery, et al., 2019) and local honey bees are hybrid populations of *A. mellifera mellifera* × *caucasica* (Requier et al., 2017). The hive entrance was fully opened during the observation period. The hornet density in the study area was considered as high (361 nests were observed in the neighboring township (Royan, equivalent to 18.7 nests/km^2^), which is more than six times higher (2.8 nests/km^2^) than the estimated population in the same area based on proportions of unobserved nests estimated from national surveys (Requier et al., 2022).

### 2.2 Flight trajectories of the Asian hornet and honey bees

The RGB‐D videos collected by the stereo‐cameras were processed using the method presented in Chiron et al. (2013) resulting in a dataset made of the trajectories of every insect flying in front of the beehive (Figure S2, Video S1). The subject of each trajectory (either Asian hornet or honey bee) was determined using both the flight dynamics such as max speed (i.e., using a clustering approach inspired by Chiron et al., 2014) as well as appearance features such as the body size. More details on size/depth ratio of targets as observed on RGB‐D images are available in Requier et al. (2016). Finally, for each trajectory, we extracted the period when it occurred (date, start time), the flight speed (m.s^−1^), the orientation (3D vector), the curvature (m), and the proportion of time spent in static flight (hovering). The Asian hornet count for each measurement was made by counting them in the 5 s before and after the measurement point. We differentiated hovering behavior into two categories: those where the individual remained very steady (threshold 1 = less than 2 mm of drifting between two images), and those where the individuals drifted slightly from their initial position (threshold 2 = less than 10 mm of drifting between two images).

### 2.3 Automated selection of interest scenes

Each video segment, for which both hornet(s) and honey bee(s) were detected, using the process just described, was automatically extracted using specifically developed software. This software included a step‐by‐step procedure composed of the following processes: (i) stereovision acquisition, (ii) target detection, in each image independently, on RGB‐D, (iii) temporal aggregation for multitarget tracking in 3D (Chiron et al., 2013), (iv) signature extraction from the individual trajectories, (v) hierarchical segmentation of the trajectory data into temporal entity, and (vi) behavioral modeling by multilevel clustering (Chiron et al., 2014). The video segments were then visually reviewed by an observer in order to detect potential successful predation of a honey bee by a hornet (Figure S3). During the analysis of the prey–predator interactions, honey bee–hornet pairs were built, and the intensity of their interaction was automatically assessed (Video S2). We considered a predation to be successful when a hornet caught a honey bee and flew out of video view with its caught prey, taking into account the limited field of view (about 1.5 m^2^ around the beehive entrance). Each video was reviewed twice by the observer to confirm the successful predation events. A predation was considered as a failure when observing both hornet(s) and honey bee(s) in the same scene but with no predation success (e.g., no catch).

### 2.4 Data analysis

All the analyses were conducted with R, version R 4.0.2 (R Core Team, 2020). The significance level for the statistical tests was set at 5% for the risk of rejecting the hypotheses.

#### 2.4.1 Daily temporal patterns of flight activities

To understand how flight performance of both honey bee and hornet vary over the time, we used Generalized Additive Models (GAMs, using the *mgcv* R‐package) of the following flight parameters: the number of trajectories per unit of time, the maximum flight speed, the flight trajectory curvature, and the percentage of time spent hovering for each flight trajectory of bees and hornets.

#### 2.4.2 Flight performance

To compare flight performances between honey bees entering or leaving and hornets, we analyzed their distribution of maximum speed, curvature, and hovering percentage. We checked for normality using an Anderson–Darling normality test (adapted for large datasets >5000 pts, using the *nortest* R‐Package) and for variance homogeneity with a Levene test (using the *car* R‐Package). We then used Kruskal–Wallis rank sum tests to assess differences in flight parameters between the three types of flight (hornets, honey bees entering, honey bees leaving the hive, followed by a pairwise Wilcoxon test as a post hoc test with a Bonferroni P value adjustment method (using the *stats* R‐Package).

#### 2.4.3 Overlapping flight performances and predation success behavior

To assess which of the global parameters best explained the hornet predation success (response variable), we ran a binomial Generalized Linear Mixed Model (GLMM, using the *GLMM* R‐Package) with fixed parameters being the number of hornets present, the hour of the day, the number of honey bees present, the interaction between all those parameters, and the quadratic parameters of hornets and hour. To select parameters of interest, we ran a multimodel inference procedure by AIC comparison (using the *MuMIn* R‐Package). In order to extract specific behavioral patterns linked with the hornet predation success, we analyzed the distribution of maximum flight speed, flight curvature, and hovering percentage for the prey (pooling honey bees entering and leaving their hive) and predator, in case of predation success or failure. To test for statistical differences between predation success and failure, we checked for variance homogeneity as described above. We then used a Kruskal–Wallis rank sum tests to assess differences in flight parameters between them, followed by a pairwise Wilcoxon test as a post hoc test with a Bonferroni *p* value adjustment method (using the *stats* R‐Package).

#### 2.4.4 Impact of hornet density on bees and hornets flight performance and predation success

To assess the impact of hornet density (Log_10_ number of hornets) on bee and hornet flight performance traits (i.e., flight speed, curvature and hovering), we used Linear Models (LM, using the *stats* R‐Package) on hornets, honey bees leaving the hive or honey bees entering the hive. Using the same statistical technique, we analyzed whether the density of hornets impacted the coefficient of variation in these three flight performance traits in hornets and in honey bees leaving the hive and entering the hive.

## 3 RESULTS

### 3.1 Time series of flight activities

**FIGURE 2:**
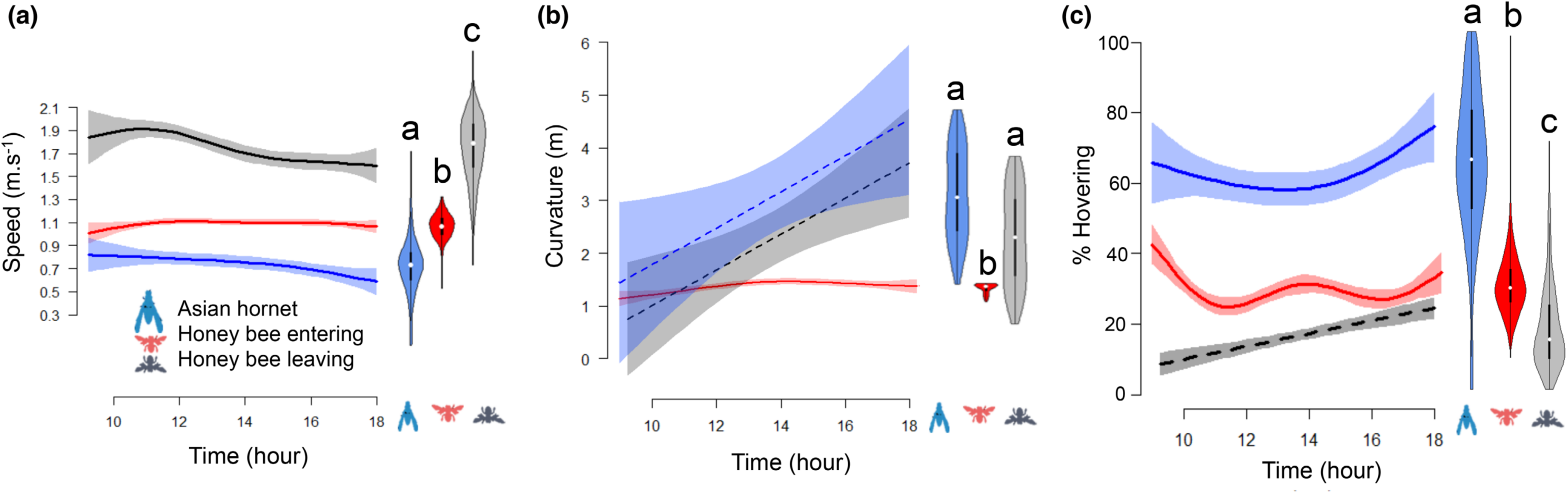
Daily time series of (a) the flight speed, (b) curvature, and (c) time spent hovering by honey bees entering (in red) or leaving (in gray) their hive and Asian hornets (in blue). Differences in letters denote significant difference using Kruskal–Wallis tests. Lines represent model predictions and shaded areas show the 95% confidence intervals. Dot lines show nonsignificant trends.

Overall, a total of 603,259 trajectories were extracted, which included 5175 predator–prey flight interactions with a total of 126 successful predation events representing 2.4% of the recorded interactions. The daily time series of flight activity in hornets and honey bees in front of the beehive followed nonlinear, quadratic patterns that increased until 1 pm for hornets (GAM, *F* = 22.9, *p* < .001) and 3 pm for honey bees (GAM, *F* = 25.95, *p* < .001) and then decreased (Figure 1b). The daily dynamic of flight speed in hornets and honey bees in front of the beehive followed nonlinear patterns with an increase until 11 am for hornets (GAM, *F* = 7.297, *p* < .001) and until 2 pm for honey bees entering the hive (GAM, *F* = 2.327, *p* < .001) and leaving the hive (GAM, *F* = 16.34, *p* < .001), and then decreased (Figure 2a).

The daily time series of flight curvature in honey bees entering the hive followed a similar nonlinear pattern to flight activity and flight speed, with an increase until 2 pm (GAM, F = 12.29, *p* < .001) and then decreased (Figure 2b). Curvatures of hornets and honey bees leaving the hive followed a positive trend over the day (GAM, *F* = 9.263, *p* = .003 and *F* = 17.03, *p* < .001, respectively). On the other hand, the daily dynamics of the percentage of time spent hovering by hornets and honey bees followed nonlinear patterns that decreased until 2 pm and then increased for hornets (GAM, *F* = 6.19, *p* < .001), and was more variable for honey bees (GAM, *F* = 13.56, *p* < .001, Figure 2c). For honey bees leaving the hive, the percentage of time spent hovering increased over the day (GAM, *F* = 58.01, *p* < .001).

### 3.2 Flight performance

**FIGURE 3:**
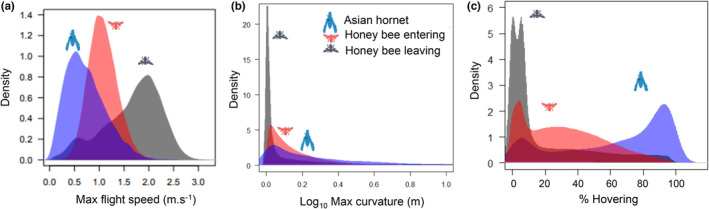
Flight performance in (a) speed, (b) curvature, and (c) time spend hovering of the Asian Hornet in blue, honey bees entering the hive in red, or leaving it in gray. Time spent hovering is based on the threshold 2 indicator (see methods).

Hornets and honey bees had different flight performances in term of flight speed, curvature and static flight (Figure 3). Honey bees leaving the hive were 1.9 times faster than honey bees entering the hive, and honey bees entering the hive were 1.25 times faster than hornets (Kruskal–Wallis chi‐squared = 78,018, *df* = 2, *p* < .001; Pairwise Wilcoxon test, *p* < .001; Figure 3a). With respect to flight curvature, the flight trajectories of honey bees leaving the hive were significantly straighter than for honey bees entering the hive, and for the latter the trajectories were significantly straighter than for hornets (Kruskal–Wallis chi‐squared = 41,384, *df* = 2, *p* < .001; Pairwise Wilcoxon test, *p* < .001). Moreover, the curvature was less variable in honey bees leaving the hive compared with the two other categories (Figure 3b).

Hornets hovered significantly for more time than honey bees (*p* < .001 (Pairwise Wilcoxon test), hornets hovered 2.1 times more than honey bees entering the hive). Honey bees leaving the hive hovered significantly less than the honey bees entering the hive (Kruskal–Wallis chi‐squared = 27,949, *df* = 2, *p* < .001; Pairwise Wilcoxon test, *p* < .001) (Figure 3c). In hovering hornets, static flight as defined by “threshold 1” (no more than 2 mm displacement) represented a very small proportion of flight time, under 10%, while if defined by “threshold 2,” up to 10 mm displacement, hovering hornets can be divided into mainly two categories. A smaller group of hornets, spending 10% of recorded flight time hovering, and a larger group of hornets spending around 90% of recorded flight time hovering, demonstrating that this kind of flight pattern is used by many for long periods of time in front of the hives (Figure S2).

### 3.3 Overlapping flight performances and predation success behavior

**FIGURE 4:**
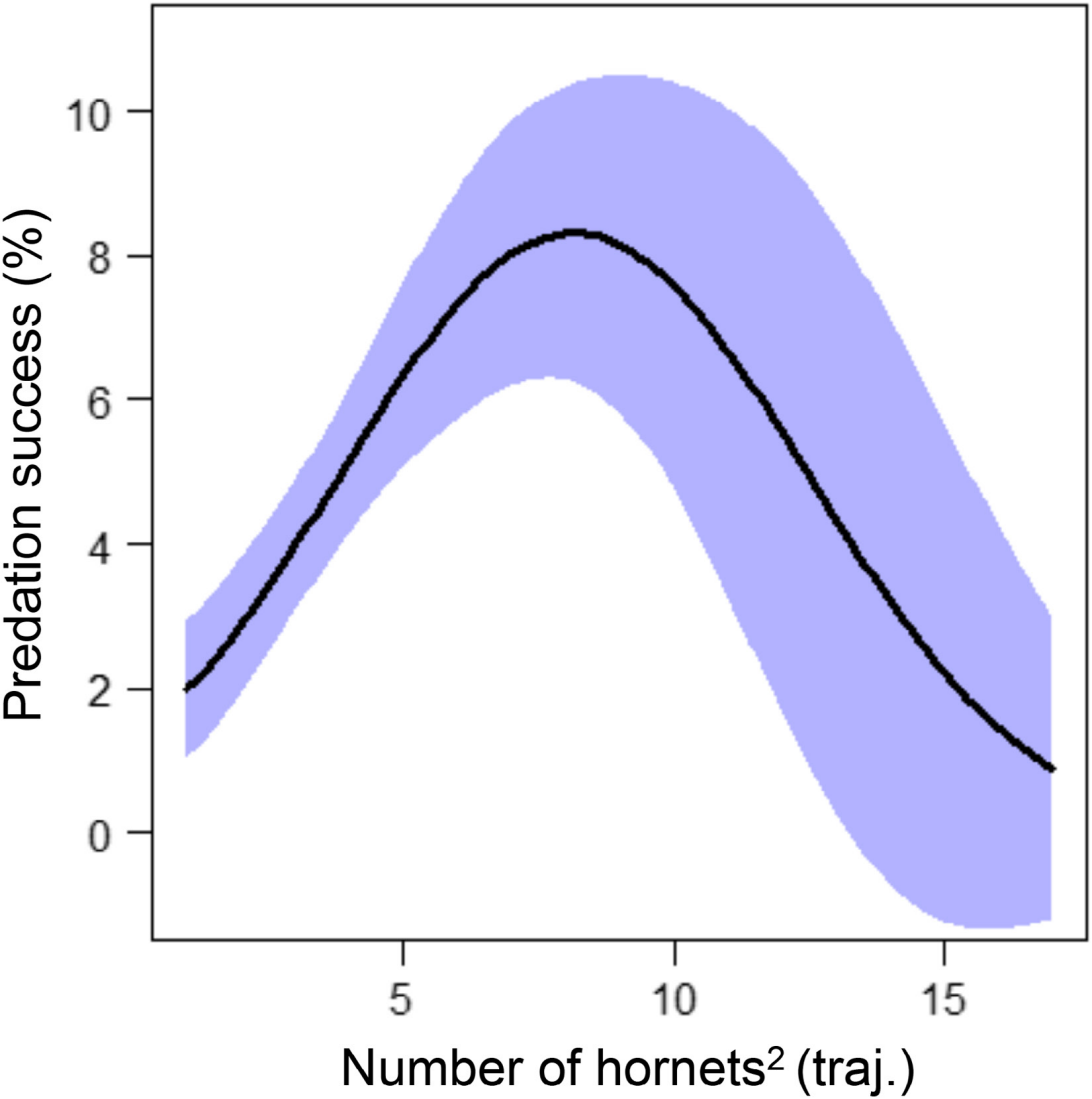
Predation success of the Asian hornets modeled as a quadratic function of hornet numbers. Line represents the model prediction and the shaded area shows the 95% confidence intervals.

When looking at predation success, this was four times higher when hornets tried to predate honey bees entering the hive (69.46% success) compared with honey bees leaving the hive (15.27% success; the remaining 15.27% predation success was attributed to bees categorized as neither entering or leaving the hive). The only global parameter that had a significant impact on hornet predation success was the number of hornets in front of the hive, when considered as a quadratic parameter (Table 1, Table S1). Predation success had a positive quadratic relationship with the square number of hornets present (Table 1) and this peaked at approximately 8 hornets (Figure 4). The number of honey bee flight trajectories, the time of day and the interactions between those parameters did not have any significant impact on hornet predation success (Table 1).

**TABLE 1:** Summary of the binomial Generalized Linear Mixed Models (GLMM) performed to assess significant parameters on the predation success of Asian hornet on honey bees.

Model parameter	Complete model estimate ± s.e.	Z	*p*‐value	Multimodel average estimate±	Relative importance
Intercept	−7.956 ± 4.954	−1.606	.1083		
Time	0.629 ± 0.743	0.848	.3964	0.149 ± 0.242	0.486
Time^2^	−0.02 ± 0.026	−0.773	.4393	−8.002 E‐03 ± 0.09	0.225
Number of honey bees	0.002 ± 0.006	0.429	.6681	−1.045 E‐04 ± 0.004	0.781
Number of hornets	0.432 ± 0.248	1.743	.0813	0.492 ± 0.045	0.781
**Number of hornets** ^**2**^	**−0.036 ± 0.008**	**−4.65**	**<.001**	**−0.035 ± 0.001**	**0.781**
Time x Number of honey bees	−0.001 ± 0.0003	−1.585	.1129	−6.104 E‐04 ± 1.431 E‐05	0.365
Time x Number of hornets	0.0001 ± 0.015	0.012	.9908	6.025 E‐04	0.042
Number of honey bees x Number of hornets	0.001 ± 0.0003	1.578	.1145	4.64E‐04 ± 3.440E‐05	0.43

*Note*: Results are given both for the complete GLMM statistical model and for a multimodel inference procedure. Detailed set of all candidate explanatory models of predation success, selected by AIC (Akaike Information Criterion) and ranked by decreasing statistical support are presented in Table S1. Bold entries indicate significant effects at a 0.05 level. Time^2^ represents a quadratic term to take into account a nonlinear pattern of the observed relationship between daily flight activity and time. The same goes for Number of hornets^2^.

We found no significant difference in hornet flight speed associated with hornet predation success or failure (Kruskal–Wallis chi‐squared = 0.44, *df* = 2, *p* = .80). In other words, the flight speed of a hornet that successfully caught a honey bee did not differ from the flight speed of those hornets that were unsuccessful. Similarly, we found no difference in percentage of time hornets spent hovering (Kruskal–Wallis chi‐squared = 0.32, *df* = 2, *p* = .85) nor with hornet flight curvature (Kruskal–Wallis chi‐squared = 1.71, *df* = 2, *p* = .43) associated with predation success or failure. Moreover, there were no significant difference in honey bee flight speed associated with hornet predation success or failure (Kruskal–Wallis chi‐squared = 2.26, *df* = 2, *p* = .32), and no significant difference in honey bee time spent hovering (Kruskal–Wallis chi‐squared = 0.93, *df* = 2, *p* = .63). But honey bee flight curvature was significantly lower in case of hornet predation success compared to unsuccessful predation attempts (Kruskal–Wallis chi‐squared = 11.10, *df* = 2, *p* = .005), meaning that the capacity of honey bees to have more curved trajectories, or less straight flight paths, allowed them to be more successful in avoiding hornet predation.

### 3.4 Impact of hornet density on bees and hornets flight performance

**FIGURE 5:**
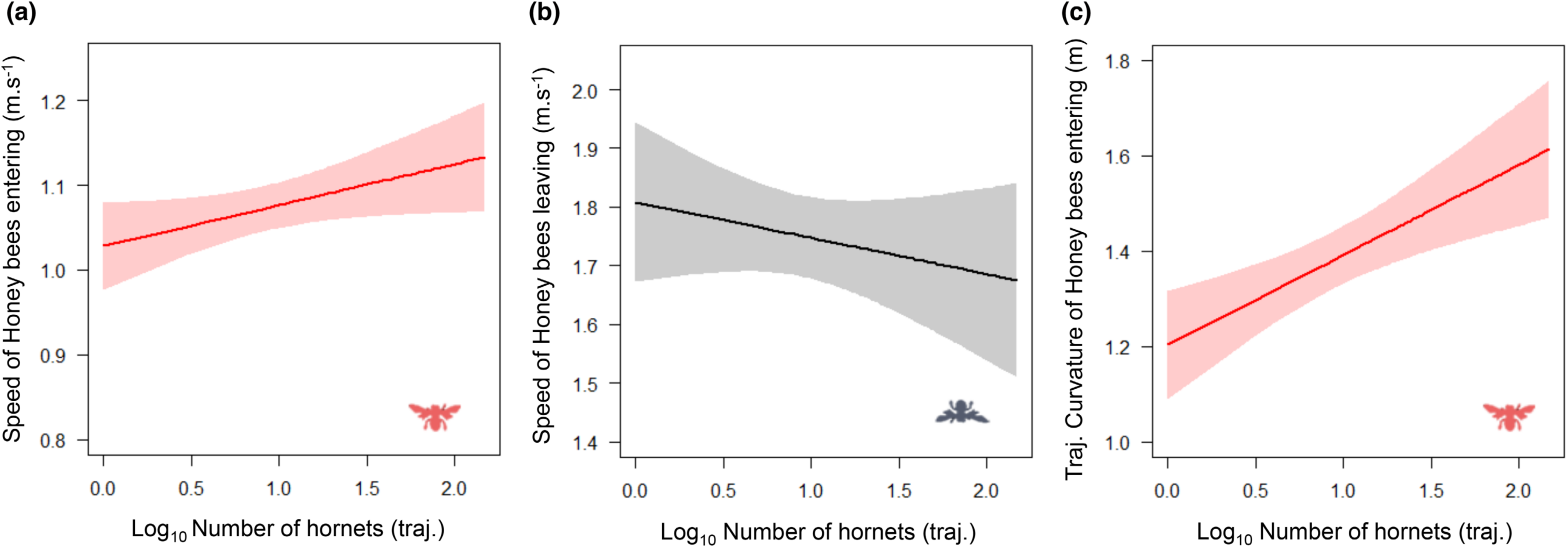
Hornet density (Log_10_ trajectories) effects on (a) speed of bees entering the hive, (b) speed of bees leaving the hive, (c) curvature of bees entering the hive trajectories. Lines represent model predictions and shaded areas show the 95% confidence intervals.

**FIGURE 6:**
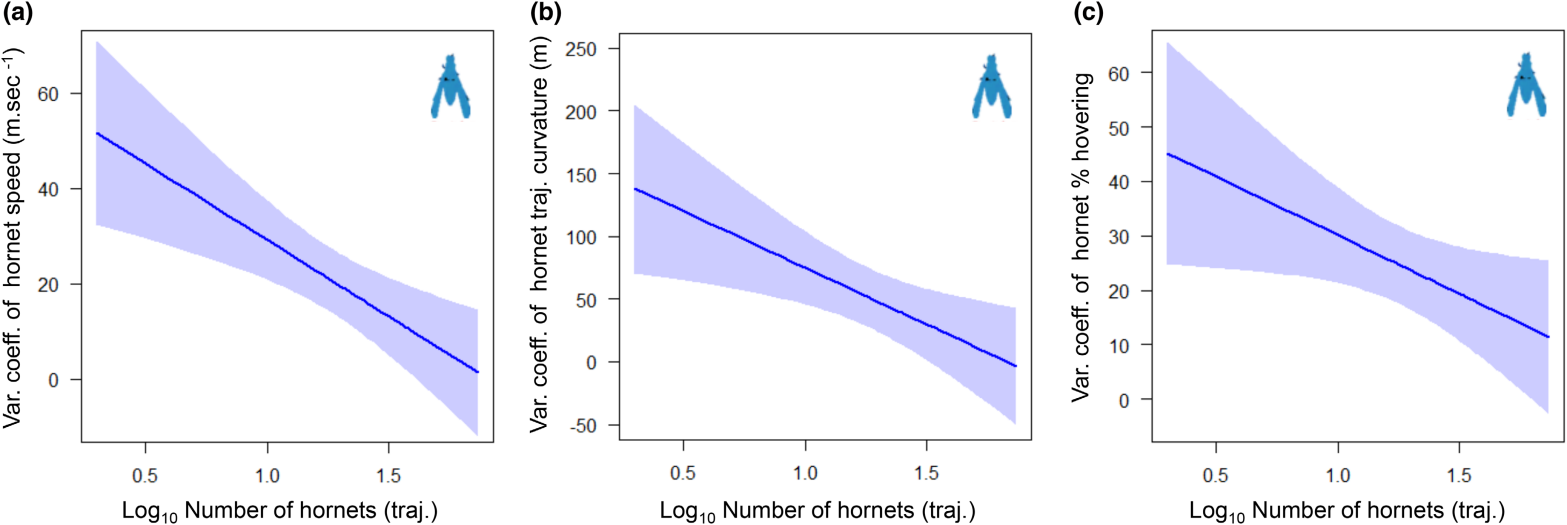
Hornet density (Log_10_ trajectories) effects on the coefficient of variation (CV) of (a) speed, (b) trajectory curvature, and (c) hovering percentage in hornets. Lines represent model predictions and shaded areas show the 95% confidence intervals.

The speed of honey bees leaving the hive was negatively affected by the number of hornets present in front of the hive (LM, *F* = 4.617, *p* = .032). However, the speed (LM, *F* = 19.36, *p* < .001) and the curvature of the trajectories of honey bees entering the hive were positively affected by the number of Asian hornets in front of the hive (LM, *F* = 59.74, *p* < .001) (Figure 5). The number of hornets in front of the hive strongly reduced the variance of all parameters (speed, curvature, and percentage of hovering) of bees entering and leaving the hive, and hornets (Figure 6), but only tend to decrease (no significant statistic) the variance of flight curvature of bees entering the hives (Table 2, Figure S5, Table S2).

**TABLE 2:** Summary of the Linear Models (LM) performed to assess impact of Asian hornet density (Log_10_ of hornet trajectories counted during 10 s) on different trajectory characteristics in honey bees and hornets.

Model parameter	Estimate *±* s.e.	*F*	*p*‐value
Bees entering the hive			
Speed	**0.0483 ± 0.011**	**19.36**	**<.001**
Curvature	**0.1888 ± 0.024**	**59.74**	**<.001**
% Hovering	0.0064 ± 0.009	0.484	.4873
Bees leaving the hive			
Speed	**−0.061 ± 0.029**	**4.617**	**.0324**
Curvature	−0.030 ± 0.370	0.007	.9356
% Hovering	0.021 ± 0.012	3.167	.0761
Hornets			
Speed	0.020 ± 0.033	0.352	.5532
Curvature	−0.693 ± 0.6116	1.285	.2580
% Hovering	0.0007 ± 0.028	0.0006	.9798

*Note*: Bold entries indicate significant effects at a 0.05 level.

## 4 DISCUSSION

In this study, we developed an automated process to select “scenes of interest,” when both prey and predator are present on the screen at the same time. Second, we explored flight performances in terms of speed, curvature, and hovering by honey bees and hornets by analyzing the 3D trajectories of the flights. Then we explored potential drivers of predation success. As hypothesized, we demonstrated that predation success was influenced by predator density, and we were able to estimate a threshold of abundance for hornets hunting in front of the hive, above which their predation success declines. Moreover, we demonstrated that hornet density had a direct impact on bee flight trajectories, shaping their flight speed and curvature.

The automatic selection of scenes of interest is a significant step forward for behavioral ecology to reduce the manual labor involved. More details on the technical part of this work were provided in Chiron et al. (2013), Chiron et al. (2014) and Requier et al. (2016). In our study, it reduced 90 observation hours into a few hours, and allowed us to switch from 603,259 registered trajectories to 5175 scenes of interest (i.e., short video segments) where both prey and predator were present. The field of view was appropriately selected, as it included the honey bees' deceleration area as they enter their colony, through its reduced entrance, that is strategically exploited by predating hornets. Such data enabled the analysis of flight performance characteristics, namely speed, curvature, and hovering (established via 3D accuracy), of honey bees and hornets, to better understand their prey–predator interaction and look for potential drivers of predation success. One perspective of this study would the potential for in‐depth analysis of the interindividual interactions among bees and hornets by placing 3D camera closer to the entrance. This could further understanding whether flight trajectories are interconnected in a more restricted area in regard to the nest entrance.

The flight speed of honey bees arriving and entering the colony was half as fast as the honey bees leaving the colony, probably because of the need of honey bees to slow down in vicinity of the nest entrance to correct their flight and enter the nest safely, that is, “parachute” behavior. To illustrate the importance of such parameter on the potential predation capacities of predators on bees, we can cite the very particular “crashing behavior” of some *Melipona* bee species. In order to prevent predation in front of their nests by spiders, while they attempt to enter their earth built nest, these bees have adapted the shape of the nest entrance into a tube with a flat platform orientated in a specific way to allow these bees to bounce on it to enter the nest quickly, without having to decrease speed, therefore, being harder to catch by predators waiting at their nest (Shackleton et al., 2019). Concerning flight curvature, honey bees leaving the hive had very straight trajectories, while honey bees entering their hive had more curved trajectories. The time spent hovering by honey bees leaving the colony was very low. Hovering seems to be a rare behavior in honey bees, linked with specific situations, while in hornets this is characteristic of their predation flight in the hive vicinity or in other “hunting sites,” for example, at other insect aggregations (bins, animal carcasses).

The flight speed of honey bees in front of the hive increased in the morning, then for honey bees leaving their hive we observed a slowing around noon, to gently decreasing through the afternoon. Hovering flights were mostly observed with hornets and returning honey bees. Hornet hovering decreased through the morning until early afternoon, and thereafter increased. This might be related to the quantity of potential prey available around the colony entrance: hornets may have to hover, to wait for predation opportunities. For the honey bees entering the hive, we observed a similar pattern, but with a slight increase in hovering in the early afternoon. This hovering activity of bees in front of the beehive could provide hornets with more opportunity to prey on them.

During the course of the day, the number of honey bees leaving their hives and hornets in front of it at any one time follows a quadratic pattern, meaning that it is enhanced through the morning up to the middle of the day, to then slowly decrease at the end of the day. The optimum period of activity for hornets was around 12 pm, while for honey bees peak activity occurred around 2 pm. This interesting offset could be linked with the predation success of hornets that decreases when there are too many hornets hovering in front of the hive. Arriving early at the hive would, even before the prey are abundant, limit the number of predating competitors. The presence and the temporal dynamics of honey bees in front of hive was very similar to what observed in Struye et al. (1994). The daily dynamics of the presence of hornets in front of the hive also matches with previous literature studying the rate at which hornets were leaving their nests over a day (Monceau et al., 2017; Poidatz et al., 2018).

Predator capture rates are expected to depend on encounter probability with prey, prey escape capability, and on predator agility (Kruse et al., 2008). We confirm that hornets succeed in preferentially catching honey bees going back into their colony (Shah & Shah, 1991): we first hypothesized that the main explanatory parameter could be their speed, which is lower than for bees leaving their colony, as they come back more slowly due to their nectar‐filled crops or pollen baskets, and need to slow down to be able to access the small hive entrance. But our analysis showed that, while returning bees were slower, the flight speed did not play a significant role in the probability of success of hornet predation. We also reveal in this study that the quadratic number of Asian hornets in front of the honey bee hive influences their predation success on honey bees, reaching a peak at 8 hornets, above which threshold their predation success decreases. In the increasing phase, there are enough active prey, and we can hypothesize that the predators are distributed at the hive entrance in a way that allows them to optimize space and occupy more and more potential honey bee paths; but above 8 hornets, hornet predation success decreases with their increasing number. It could be due to interspecific competition (Monceau et al., 2014), and maybe also be due to the foraging paralysis of honey bees, that do not exit the colony at the same rate anymore.

This result is congruent with the results of the escape success in terrestrial predator–prey interactions model developed by Wilson et al. (2020), where those authors concluded that smaller prey with higher agility would force larger predators to run along curved paths that do not allow them to use their superior speeds, and therefore could be a critical parameter for escaping predation. Moreover, in their study of the goshawk's *Accipiter gentilis* predation, Kane et al. (2015) showed a similar conclusion: the prey's sharp sideways turns caused the goshawk to lose visual fixation on the prey and thus decreased their predation success.

The increasing number of hornets present in front of the hive strongly affected both hornet behavior and honey bee behavior on entering and leaving their hive. First, an increased number of hornets reduced the speed of bees leaving the hive, suggesting more hesitancy from bees going out to forage. The number of hornets also enhanced the speed and the trajectory curvature of honey bees entering the hive, so the bees are ‘racing’ into the hive, and choosing an unpredictable flight path—both of which may reduce their chances of capture. Very interestingly, this result fits with the adaptive behavior of *Apis cerana* foragers to escape *V. velutina* predation (Tan et al., 2007). *Apis cerana* are native honey bees from Asia and coexist with *V. velutina* in this region where both the Asian honey bee and Asian hornet are native. The similar behavior of bees from Europe (our study) and Asia in the presence of an abundance of hornet predators suggests that increasing flight speed of honey bees entering the hive, that is, the preferential prey of hornets in comparison with honey bees leaving their hive, would improve bee survival and limit hornet predation success. Second, increasing the number of hornets reduced the variance in flight patterns for bees and hornets. This could be advantageous to hornets at first as they have a lower range of bee flight trajectories to tackle and anticipate for predation success.

Although based on a single beehive, this study highlights the potential of such observations and analytical methods, as they provided unique and useful data that allowed the observer to accurately witness complex phenomena congruent with the literature, and provided interesting leads for further studies. The automatic processing method providing “Filtered video sequences of potential predation” from “RGB‐D sequences” represents a very useful tool for video‐based data collection in ecology. Some improvement points can be recommended for future studies. External uncontrolled events (e.g., other flying insects coming into the field of view of the video camera, extreme weather episodes, overcrowded flight area) are likely to induce biases in the final statistics for the following reasons: target miss detections, failure during tracking, and erroneous built‐in depth estimation by the stereo‐camera. Therefore, relative analysis and examination of trends is more conservative. For absolute figures, for example, of “hornet predation success rate,” the data would benefit from confirmation with complementary studies. This method could also enable deeper description of such behaviors: for example, through marking hornet hunters of different ages (differing in hunting experience) and colonies, it could be possible to describe individual learning, and to detect behavioral differences between colonies. In the same way, through marking escaped bees, their learning capacity could be further evaluated.

In summary, 3D flight analysis of both predator and prey has demonstrated the characteristics of the bees' flight that change (speed and trajectory curvature) as predatory pressure at the hive increases, suggesting avoidance behavior. It has also shown that curvature of the bees' flight has more effect than flight speed on hornet predatory success. As honey bee colonies in Europe are now under considerable pressure from this predator, it would be of great interest for future work to focus on whether different bee colonies show different flight behaviors making them more or less resilient to attack by this voracious predator.

## AUTHOR CONTRIBUTIONS

**Juliette Poidatz:** Formal analysis (supporting); writing – original draft (equal). **Guillaume Chiron:** Conceptualization (equal); data curation (lead); software (lead); writing – review and editing (supporting). **Peter Kennedy:** Writing – review and editing (equal). **Juliet Osborne:** Writing – review and editing (equal). **Fabrice Requier**: Conceptualization (equal); Formal analysis (lead); writing – original draft (equal).

## CONFLICT OF INTEREST STATEMENT

The authors reported no potential conflict of interest.

## Supporting information


Data S1.

Video S1.

Video S2.

## Data Availability

The data that support the findings of this study is openly available through the figshare repository https://doi.org/10.6084/m9.figshare.22233067.v1 (Poidatz et al., 2023).
