# Supplementary material for: Density of predating Asian hornets at hives disturbs the 3D flight performance of honey bees and decreases predation success
Source: Ecol Evol. 2023 Mar 28;13(3):e9902. doi: 10.1002/ece3.9902 (PMC10049882; doi:10.1002/ece3.9902)
Supplement: Supplementary file 1 — Data S1. [file ECE3-13-e9902-s001.docx]

**Density of predating Asian hornets at hives disturbs the 3D flight performance of honey bees and decreases predation success**

*Poidatz J., Chiron G., Kennedy P., Osborne J., Requier F.*

**ELECTRONIC SUPPORTING INFORATION**

| **Content** |  | **pj** |
| --- | --- | --- |
| Figure S1 | Illustration of the stereovision camera. | 2 |
| Figure S2 | Top view of the honey bees trajectories. | 3 |
| Figure S3 | Interest scene selection process. | 4 |
| Figure S4 | Density polygons of the percentage of time spent hovering by Asian Hornets separated by threshold used. | 5 |
| Figure S5 | Hornet density (Log_10_ trajectories) effects on honey bee flight parameters. | 6 |
| Table S1 | Detailed set of all candidate explanatory models of predation success. | 7 |
| Table S2 | Summary of the Linear Models (LM) performed to assess impact of Asian hornet density. | 10 |
| Video S1 | Building process of the trajectometry. | 11 |
| Video S2 | Interaction and predation events automatic scene selection. | 11 |

**Figure S1.** Illustration of the stereovision camera (G3 Evo 3, TYZX®) (a), and detailed disposition of the camera on the top of the hive (b).

**
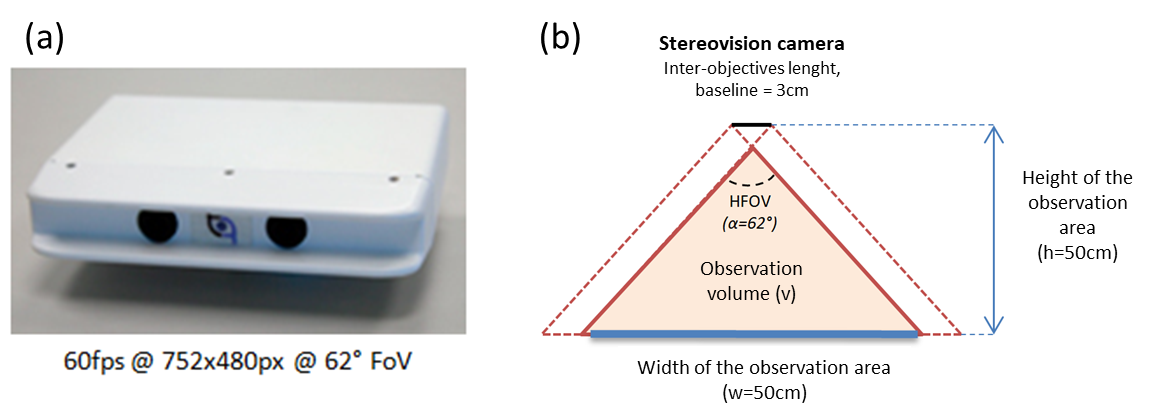
**

**Figure S2.** Top view of the honey bees trajectories, each coloured line being an individual trajectory, associated with an individual label. (a), depth adding to the individual positions (b), individual 3D trajectometry building (c).

**
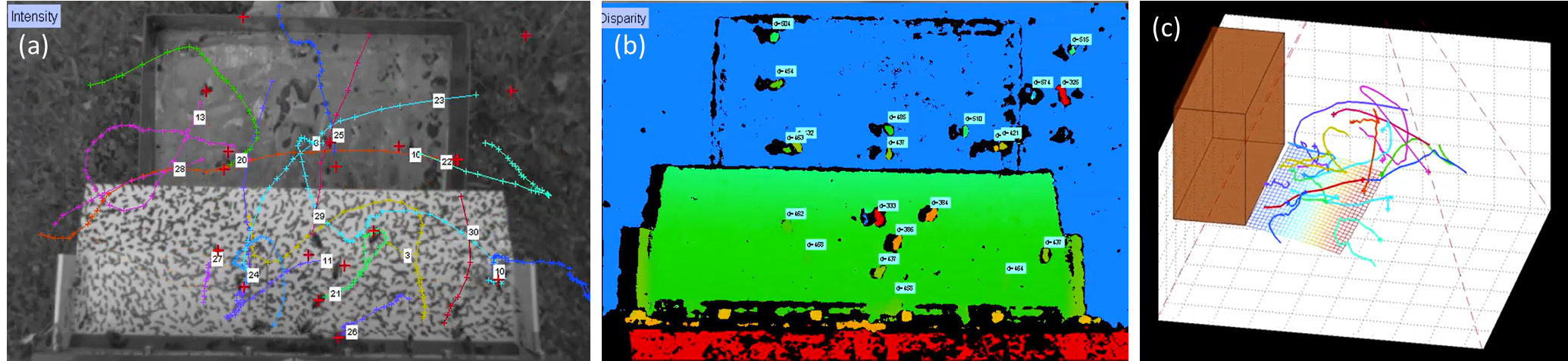
**

**Figure S3.** Interest scene selection process: automatized selection of video sections where both bees (in blue) and hornets (in orange) are spotted at the same time. The selected videos were then visually analysed to detect whether they were predation cases and their result.

**Figure S4**. Density polygons of the percentage of time spent hovering by Asian Hornets separated by threshold used. Threshold 1 (in red) allows less than 2mm of drifting between 2 images, whereas the threshold 2 (in blue) allows the individuals to slightly drift from their initial position with less than 10mm of drifting between 2 images.

**Figure S5.** Hornet density (Log_10_ trajectories) effects on coefficient of variation of (a) speed, (b) trajectory curvature and (c) hovering percentage in honey bees entering the hive, and on coefficient of variation of (d) speed, (e) trajectory curvature and (f) hovering percentage in honey bees leaving the hive. Lines represent model predictions and shaded areas show the 95% confidence intervals.

**Table S1.** Detailed set of all candidate explanatory models of predation success, selected by AIC (Akaike Information Criterion) and ranked by decreasing statistical support. AIC is an inverse indicator of model parsimony considering fit (logLik = log-Likelihood) and complexity (k = number of parameters to be estimated in the candidate model). Estimates are given for each explanatory variable after those were centred to mean = 0 and standardized to sd. =0.5. Models ignoring the dose effect all received a virtually negligible statistical support (AIC weight of evidence w_i_<0.001, that is, the probability of model i as being the best model in the set is nearly 0). The top-model set with a ∆AIC <2 (AIC difference with the best candidate model) comprises four concurrent models (ranks 1–11, in bold) with a weight of evidence w_i_ ranging from about 12 to 28%. All environmental correlates of honey bee navigation and their interactions with the dose effect are retained in at least one of the top models. The ∆AIC <2 cut-off rule was used to define the top-model set

| Model rank | Intercept | hour | hour^2 | Honey bees number | Hornet number | Hornet number^2 | hour x honey bee number | hour x hornet number | Honey bee x hornet numbers | df | logLik | AIC | delta AIC | weight |
| --- | --- | --- | --- | --- | --- | --- | --- | --- | --- | --- | --- | --- | --- | --- |
| 190 | **-4.264** | **0.06111** |  | **0.00363** | **0.4504** | **-0.036** | **-0.0006083** |  | **4.51E-04** | **7** | **-994.667** | **2003.3** | **0** | **0.113** |
| 62 | **-4.642** | **0.05974** |  | **0.005914** | **0.5284** | **-0.03365** | **-0.0006255** |  |  | **6** | **-995.689** | **2003.4** | **0.05** | **0.111** |
| 157 | **-3.465** |  |  | **-0.004908** | **0.4645** | **-0.03634** |  |  | **4.75E-04** | **5** | **-996.853** | **2003.7** | **0.37** | **0.094** |
| 29 | **-3.89** |  |  | **-0.002742** | **0.5484** | **-0.03399** |  |  |  | **4** | **-998.075** | **2004.2** | **0.82** | **0.075** |
| 159 | **-3.241** |  | **-0.001299** | **-0.004695** | **0.4657** | **-0.03617** |  |  | **4.42E-04** | **6** | **-996.231** | **2004.5** | **1.13** | **0.064** |
| 31 | **-3.598** |  | **-0.001474** | **-0.002684** | **0.5425** | **-0.03393** |  |  |  | **5** | **-997.277** | **2004.6** | **1.22** | **0.062** |
| 158 | **-3.02** | **-0.03526** |  | **-0.004646** | **0.4667** | **-0.03616** |  |  | **4.38E-04** | **6** | **-996.28** | **2004.6** | **1.23** | **0.061** |
| 30 | **-3.333** | **-0.04079** |  | **-0.002644** | **0.5428** | **-0.03394** |  |  |  | **5** | **-997.309** | **2004.6** | **1.28** | **0.06** |
| 192 | **-7.966** | **0.6306** | **-0.02024** | **0.002384** | **0.4346** | **-0.03633** | **-0.0005875** |  | **5.29E-04** | **8** | **-994.365** | **2004.7** | **1.4** | **0.056** |
| 64 | **-6.32** | **0.313** | **-0.008996** | **0.005541** | **0.5276** | **-0.03363** | **-0.0006178** |  |  | **7** | **-995.625** | **2005.3** | **1.92** | **0.043** |
| 254 | **-4.237** | **0.05919** |  | **0.0037** | **0.4417** | **-0.03593** | **-0.0006131** | **0.0006025** | **4.51E-04** | **8** | **-994.666** | **2005.3** | **2** | **0.042** |
| 126 | -4.598 | 0.05663 |  | 0.006001 | 0.5155 | -0.03356 | -0.0006316 | 0.0008898 |  | 7 | -995.687 | 2005.4 | 2.04 | 0.041 |
| 160 | -7.689 | 0.68 | -0.0253 | -0.005835 | 0.4451 | -0.03652 |  |  | 5.37E-04 | 7 | -995.783 | 2005.6 | 2.23 | 0.037 |
| 222 | -3.512 | 0.001655 |  | -0.004758 | 0.5817 | -0.03705 |  | -0.008194 | 4.55E-04 | 7 | -996.116 | 2006.2 | 2.9 | 0.027 |
| 32 | -5.953 | 0.3536 | -0.01397 | -0.003048 | 0.5406 | -0.03388 |  |  |  | 6 | -997.146 | 2006.3 | 2.96 | 0.026 |
| 94 | -3.736 | -0.01138 |  | -0.002672 | 0.6342 | -0.03452 |  | -0.006375 |  | 6 | -997.208 | 2006.4 | 3.08 | 0.024 |
| 256 | -7.956 | 0.6297 | -0.02022 | 0.002406 | 0.4321 | -0.03631 | -0.000589 | 0.0001784 | 5.29E-04 | 9 | -994.365 | 2006.7 | 3.4 | 0.021 |
| 224 | -8.199 | 0.7192 | -0.02537 | -0.005954 | 0.5616 | -0.03744 |  | -0.008303 | 5.55E-04 | 8 | -995.621 | 2007.2 | 3.91 | 0.016 |
| 128 | -6.276 | 0.3094 | -0.008966 | 0.005617 | 0.5163 | -0.03356 | -0.0006231 | 0.0007791 |  | 8 | -995.624 | 2007.2 | 3.91 | 0.016 |
| 96 | -6.294 | 0.3743 | -0.01369 | -0.003068 | 0.6295 | -0.03445 |  | -0.006195 |  | 7 | -997.052 | 2008.1 | 4.77 | 0.01 |
| 28 | 4.951 | -1.314 | 0.04463 |  | 0.5274 | -0.03286 |  |  |  | 5 | -1002.57 | 2015.1 | 11.81 | 0 |
| 92 | 4.768 | -1.309 | 0.04522 |  | 0.5941 | -0.03329 |  | -0.004638 |  | 6 | -1002.52 | 2017 | 13.7 | 0 |
| 26 | -3.525 | -0.06536 |  |  | 0.5045 | -0.03175 |  |  |  | 4 | -1006 | 2020 | 16.67 | 0 |
| 27 | -4.023 |  | -0.002079 |  | 0.5045 | -0.03171 |  |  |  | 4 | -1006.34 | 2020.7 | 17.35 | 0 |
| 25 | -4.456 |  |  |  | 0.513 | -0.03177 |  |  |  | 3 | -1007.67 | 2021.3 | 18.01 | 0 |
| 90 | -3.607 | -0.0595 |  |  | 0.5226 | -0.03187 |  | -0.001256 |  | 5 | -1006 | 2022 | 18.67 | 0 |
| 46 | -3.7 | 0.06257 |  | 0.006767 | 0.1018 |  | -0.0006695 |  |  | 5 | -1009.51 | 2029 | 25.69 | 0 |
| 110 | -3.086 | 0.01518 |  | 0.008525 | -0.1015 |  | -0.0007922 | 0.01531 |  | 6 | -1008.77 | 2029.5 | 26.2 | 0 |
| 13 | -2.901 |  |  | -0.002495 | 0.1182 |  |  |  |  | 3 | -1012.23 | 2030.5 | 27.13 | 0 |
| 15 | -2.6 |  | -0.001547 | -0.002425 | 0.113 |  |  |  |  | 4 | -1011.35 | 2030.7 | 27.37 | 0 |
| 14 | -2.324 | -0.04261 |  | -0.002382 | 0.1132 |  |  |  |  | 4 | -1011.4 | 2030.8 | 27.46 | 0 |
| 48 | -5.76 | 0.3738 | -0.01107 | 0.006273 | 0.1014 |  | -0.0006581 |  |  | 6 | -1009.42 | 2030.8 | 27.5 | 0 |
| 174 | -3.665 | 0.06252 |  | 0.006581 | 0.09337 |  | -0.0006666 |  | 3.52E-05 | 6 | -1009.51 | 2031 | 27.68 | 0 |
| 112 | -5.043 | 0.3111 | -0.01053 | 0.008044 | -0.1018 |  | -0.0007807 | 0.0153 |  | 7 | -1008.68 | 2031.4 | 28.03 | 0 |
| 238 | -3.009 | 0.01471 |  | 0.008233 | -0.1249 |  | -0.0007932 | 0.01574 | 7.36E-05 | 7 | -1008.73 | 2031.5 | 28.13 | 0 |
| 16 | -5.516 | 0.4381 | -0.01704 | -0.002889 | 0.1118 |  |  |  |  | 5 | -1011.15 | 2032.3 | 28.96 | 0 |
| 141 | -2.81 |  |  | -0.002881 | 0.09578 |  |  |  | 9.26E-05 | 4 | -1012.17 | 2032.3 | 29.01 | 0 |
| 78 | -1.898 | -0.07547 |  | -0.002335 | 0.01465 |  |  | 0.007492 |  | 5 | -1011.2 | 2032.4 | 29.07 | 0 |
| 143 | -2.552 |  | -0.001519 | -0.002653 | 0.09979 |  |  |  | 5.47E-05 | 5 | -1011.33 | 2032.7 | 29.33 | 0 |
| 142 | -2.285 | -0.04179 |  | -0.002598 | 0.1008 |  |  |  | 5.14E-05 | 5 | -1011.38 | 2032.8 | 29.42 | 0 |
| 176 | -6.014 | 0.4231 | -0.01283 | 0.005806 | 0.0837 |  | -0.0006502 |  | 7.32E-05 | 7 | -1009.38 | 2032.8 | 29.43 | 0 |
| 240 | -5.415 | 0.3844 | -0.01317 | 0.007474 | -0.1374 |  | -0.0007792 | 0.01594 | 1.13E-04 | 8 | -1008.6 | 2033.2 | 29.87 | 0 |
| 80 | -5.172 | 0.4196 | -0.01762 | -0.002856 | 0.007242 |  |  | 0.007943 |  | 6 | -1010.93 | 2033.9 | 30.53 | 0 |
| 144 | -5.91 | 0.5116 | -0.01958 | -0.003424 | 0.08539 |  |  |  | 1.09E-04 | 6 | -1011.07 | 2034.1 | 30.81 | 0 |
| 206 | -1.851 | -0.07482 |  | -0.002578 | -0.0005903 |  |  | 0.007596 | 5.78E-05 | 6 | -1011.18 | 2034.4 | 31.02 | 0 |
| 208 | -5.59 | 0.4988 | -0.02039 | -0.003437 | -0.0251 |  |  | 0.008224 | 1.18E-04 | 7 | -1010.84 | 2035.7 | 32.35 | 0 |
| 12 | 4.643 | -1.12 | 0.03775 |  | 0.1101 |  |  |  |  | 4 | -1015.92 | 2039.8 | 36.5 | 0 |
| 76 | 4.889 | -1.118 | 0.0362 |  | -0.01354 |  |  | 0.009411 |  | 5 | -1015.62 | 2041.2 | 37.9 | 0 |
| 54 | -3.603 | 0.0769 |  | 0.009359 |  | 0.004658 | -0.0008413 |  |  | 5 | -1016.1 | 2042.2 | 38.87 | 0 |
| 10 | -2.534 | -0.06606 |  |  | 0.1026 |  |  |  |  | 3 | -1018.49 | 2043 | 39.64 | 0 |
| 11 | -3.031 |  | -0.002137 |  | 0.103 |  |  |  |  | 3 | -1018.79 | 2043.6 | 40.25 | 0 |
| 56 | -6.103 | 0.4545 | -0.01343 | 0.00876 |  | 0.004635 | -0.0008279 |  |  | 6 | -1015.96 | 2043.9 | 40.58 | 0 |
| 74 | -1.764 | -0.1242 |  |  | -0.06925 |  |  | 0.01311 |  | 4 | -1017.96 | 2043.9 | 40.59 | 0 |
| 9 | -3.472 |  |  |  | 0.1105 |  |  |  |  | 2 | -1020.22 | 2044.4 | 41.1 | 0 |
| 23 | -2.202 |  | -0.001976 | -0.002177 |  | 0.005609 |  |  |  | 4 | -1019.12 | 2046.2 | 42.91 | 0 |
| 22 | -1.85 | -0.05436 |  | -0.002123 |  | 0.005629 |  |  |  | 4 | -1019.2 | 2046.4 | 43.06 | 0 |
| 21 | -2.566 |  |  | -0.002276 |  | 0.006065 |  |  |  | 3 | -1020.58 | 2047.2 | 43.82 | 0 |
| 24 | -5.935 | 0.5604 | -0.0218 | -0.002777 |  | 0.005525 |  |  |  | 5 | -1018.78 | 2047.6 | 44.22 | 0 |
| 38 | -3.749 | 0.09409 |  | 0.01243 |  |  | -0.001049 |  |  | 4 | -1020.09 | 2048.2 | 44.84 | 0 |
| 40 | -6.577 | 0.5209 | -0.01517 | 0.01177 |  |  | -0.001035 |  |  | 5 | -1019.9 | 2049.8 | 46.46 | 0 |
| 20 | 3.843 | -0.9398 | 0.03093 |  |  | 0.005421 |  |  |  | 4 | -1023.27 | 2054.5 | 51.2 | 0 |
| 18 | -2.032 | -0.07774 |  |  |  | 0.005041 |  |  |  | 3 | -1025.08 | 2056.2 | 52.82 | 0 |
| 7 | -1.972 |  | -0.002623 | -0.001909 |  |  |  |  |  | 3 | -1025.2 | 2056.4 | 53.06 | 0 |
| 6 | -1.502 | -0.07221 |  | -0.001836 |  |  |  |  |  | 3 | -1025.32 | 2056.6 | 53.31 | 0 |
| 19 | -2.601 |  | -0.002592 |  |  | 0.005055 |  |  |  | 3 | -1025.4 | 2056.8 | 53.46 | 0 |
| 8 | -6.625 | 0.6986 | -0.02735 | -0.002659 |  |  |  |  |  | 4 | -1024.64 | 2057.3 | 53.94 | 0 |
| 17 | -3.11 |  |  |  |  | 0.005663 |  |  |  | 2 | -1027.55 | 2059.1 | 55.77 | 0 |
| 5 | -2.435 |  |  | -0.002047 |  |  |  |  |  | 2 | -1027.85 | 2059.7 | 56.37 | 0 |
| 4 | 2.812 | -0.7485 | 0.0235 |  |  |  |  |  |  | 3 | -1028.92 | 2063.8 | 60.51 | 0 |
| 2 | -1.646 | -0.09472 |  |  |  |  |  |  |  | 2 | -1030.03 | 2064.1 | 60.72 | 0 |
| 3 | -2.322 |  | -0.003247 |  |  |  |  |  |  | 2 | -1030.35 | 2064.7 | 61.37 | 0 |

**Table S2**. Summary of the Linear Models (LM) performed to assess impact of Asian hornet density (Log_10_ of hornet trajectories counted during 10 seconds) on the coefficient of variation of different trajectory characteristics in honey bees and hornets.

| Model parameter | Estimate ± s.e. | F | P-value |
| --- | --- | --- | --- |
| *Bees entering the hive* |  |  |  |
| Speed | **-5.924 ± 1.293** | **21.01** | **<0.001** |
| Curvature | -6.223 ± 3.241 | 3.686 | 0.062 |
| % Hovering | **-16.763 ± 4.157** | **16.26** | **<0.001** |
| *Bees leaving the hive* |  |  |  |
| Speed | **-7.074 ± 1.993** | **12.6** | **0.001** |
| Curvature | **-62.33 ± 19.82** | **9.895** | **0.003** |
| % Hovering | **-44.343 ± 7.063** | **39.41** | **<0.001** |
| *Hornets* |  |  |  |
| Speed | **-32.097 ± 4.367** | **54.02** | **<0.001** |
| Curvature | **-90.20 ± 15.28** | **34.85** | **<0.001** |
| % Hovering | **-21.521 ± 4.627** | **21.63** | **<0.001** |

**Video S1.** Building process of the trajectometry, with, from the left to the right, video recording, depth adding and trajectometry building.

**Video S2.** Interaction and predation events automatic scene selection. Honey bees are marked with a red cross, hornets with a blue cross. In the top part of the video, the white and grey scale illustrates the “intensity” of the interaction between hornets and honey bees, the darker the colour is, the more intense the predation event is. A major convergence of a honey bee – hornet pair is notified by a blue bar in the scale. Below is a record of different selected scenes where hornets and honey bees were present at the same time, the red line represents a honey bee – hornet pair with a potential predation event. Video recorded on the 24/10/2013 – 12:18:29.
